# Notes

**Published:** 1900-05

**Authors:** 


### NOTES.

Meat-inspectors in the Bureau of Animal Industry, appointed under the Civil Service Commission, July, 1898, to June, 1899: Drs. Joseph O. Jacobs, S. Keane, California; Michael T. Naughton, Charles W. Johnson, Howard M. Batchelder, John A. Sloan, Illinois; Hugh M. Rowe, James L. Otterman, Iowa; Herbert Caldwell, Kentucky; M. Smith, Kansas; Albert Long, Maine; Alexander E. Wight, Massachusetts; George T. Irons, Michigan; Tait Butler, Mississippi; Robert A. Ramsey, Missouri; Thomas H. Ripley, Adolph J. Pistor, Richard W. Hewett, Archibald H. Wallace, New Jersey; Daniel G. Shumway, Monroe B. Miller, Joseph Thackaberry, New York; Harry M. Ball, Levi P. Beechy, John S. Grove, George W. Butler, Ohio; George Jobson, William G. Shaw, Eli L. Bertram, John H. McNeall, Pennsylvania; Joseph M. Good, Tennessee.

Dr. Harry A. Meisner, of Baltimore, Md., as forecasted in this JOURNAL, has been appointed State Veterinarian, succeeding Dr. A. W. Clement. Dr. Meisner is a graduate of the Veterinary Department, University of Pennsylvania, and has been in active practice in Baltimore for some ten years. He is well equipped for the position, and will no doubt render efficient service and bring his State in line with all others in sanitary control work.

